# Spot-On Skin Lipid Complex as an Adjunct Therapy in Dogs with Atopic Dermatitis: An Open Pilot Study

**DOI:** 10.4061/2011/281846

**Published:** 2011-09-29

**Authors:** Masato Fujimura, Yoshinobu Nakatsuji, Subaru Fujiwara, Christophe Rème, Hugues Gatto

**Affiliations:** ^1^Fujimura Animal Allergy Hospital, Aomatanihigashi 5-10-26, Minou-shi, Osaka 562-0022, Japan; ^2^Virbac Japan, New Awajimachi Building 6F, 1-3-14 Awaji-machi, Chuo-ku, Osaka 541-0047, Japan; ^3^Virbac Japan, Kanto Office, Sumisei Shinyokohama 2nd Building 7F, 3-18-14 Shinyokohama, Kohoku-ku, Yokohama-shi, Kanagawa 222-0033, Japan; ^4^Virbac SA, BP27, 06511 Carros Cedex, France

## Abstract

The purpose of this paper was to evaluate the efficacy of topical skin lipid complex (SLC) in canine atopic dermatitis (AD). Eight dogs with chronic AD and no improvement of main therapy in symptoms, erythema, lichenification, excoriation, and alopecia in the previous month were treated with SLC topically as adjunct therapy at lesion sites twice weekly for 12 weeks. A statistically significant reduction (26.0%, *P* < 0.05) in the third version of the Canine Atopic Dermatitis Extent and Severity Index (CADESI-03) modification from baseline was recorded 6 weeks after treatment, with marked reduction in the erythema subscore (36.2%, *P* < 0.005). A significant reduction in excoriation and alopecia subscores was observed 6 weeks after treatment (39.9%, *P* < 0.05 and 19.9%, *P* < 0.05, resp.). However, the lichenification subscore was not reduced significantly at 6 or 12 weeks. These findings suggest that topical SLC may have therapeutic and clinical benefits in dogs with AD.

## 1. Introduction

Atopic dermatitis (AD) is a common allergic skin disease in both people and dogs. The clinical signs of canine AD are variable, and the diagnosis of canine AD is made by eliminating other causes of pruritus; the diagnosis is one of exclusion [1]. The treatment of dogs with AD is complex and lifelong and involves multiple modalities depending on the severity of clinical signs, the dog's response to medical therapy, and whether clinical signs are seasonal or perennial [1]. In people with AD, a study has shown impairment of the skin barrier function and abnormal lipid production [2]. In one human study, the application of a topical moisturizer containing ceramides produced dramatic improvement in clinical scores after 3 and 6 weeks of treatment [3]. In dogs with AD, electron microscopy (EM) revealed sparse and disorganized lamellar lipids (LLs) and deficiencies of the stratum corneum intercellular lipids [4]. This finding suggests that similar defects or abnormalities of the skin barrier may be involved in the pathogenesis of canine AD. In dogs with AD, the application of a topical skin lipid complex (SLC) led to improvements in intercorneocyte structure, production of LLs, and filling of intercorneocyte spaces with newly formed LLs, as shown by EM [5]. In another study, it was suggested that decreased amounts of ceramides and increased cholesterol content may be involved in epidermal barrier dysfunction [4]. In addition, correlations were found between transepidermal water loss and relative ceramide content in both lesional and nonlesional skin of dogs with AD [6]. ALLERDERM Spot-On (Virbac SA, Carros, France) is a topical spot-on skin lipid therapy that contains ceramides, cholesterol, and free fatty acids. The goal of this study was to evaluate the clinical benefits of topical SLC in the treatment of dogs with chronic AD by using the third version of the Canine Atopic Dermatitis Extent and Severity Index (CADESI-03) modification.

## 2. Materials and Methods

### 2.1. Animals

The investigators recruited 8 dogs from their hospital with a diagnosis of nonseasonal AD and persistent pruritus despite therapy. The breed, sex, age, and age of disease onset of the 8 dogs are given in Table 1. The age of the dogs at entry in the study was 2–12 years (mean: 6.75 years). The age of disease onset was 1–3 years (mean: 1.75 years). Two female dogs, 5 spayed female dogs, and 1 castrated male dog were included in the study. This open clinical trial was conducted between September 2009 and December 2009 with the owner's informed consent.

### 2.2. Pretreatment Assessment

The diagnosis of AD was made by ruling out other causes of pruritus. Dogs were negative for demodicosis based upon negative skin scrapings. The dogs underwent rigorous flea control and appropriate treatment for contagious parasitic mites. Bacterial pyoderma and yeast overgrowth (*Malassezia* dermatitis) were treated, if present. Finally, all dogs underwent an elimination diet using “hypoallergenic” foods (Hill's prescription diet canine z/d Ultra: Hill's Pet Nutrition, Topeka, KS, USA; or Royal Canin Veterinary Diet Sensitivity Control: Royal Canin, Aimargue, France; or Iams Veterinary Formulas FP: Cincinnati, Ohio, USA) for at least 8 weeks with no improvement. The diagnosis of AD was based upon elimination of other causes of pruritus, compatible history, and compatible clinical signs, as well as Willemse's criteria for the diagnosis of nonseasonal AD [7]. Intradermal allergy testing was performed with 24 selected antigens (Greer Pharmacy, Lenoir, NC, USA; or TORII Pharmaceutical co., Ltd., Tokyo, Japan). Seven dogs had positive reactions to house dust mite mix (**Dermatophagoides* farinae* and *D. pteronyssinus*) and 1 dog had positive reactions to cotton (Table 1).

### 2.3. SLC

A topical SLC product containing ceramides, cholesterol, and free fatty acids (ALLERDERM Spot-On; Virbac SA, Carros, France) was applied at a dose of 2 mL twice weekly, by applying several drops on the head, interscapular area, and directly on lesion sites.

### 2.4. Concurrent Treatments

Allergen-specific immunotherapy and/or immunomodulating drugs such as pentoxifylline were continued as a combination therapy for Dogs 1–7 (Table 1). Seven dogs were receiving allergen-specific immunotherapy for 6 to 36 months prior to inclusion in the study. Three of these 7 dogs were also receiving long-term oral pentoxifylline for the management of pruritus. Assessments were performed 4 weeks prior to starting the study to ensure that there was no improvement in clinical signs from any concurrent therapies. No other therapies (e.g., corticosteroids, cyclosporine, and antimicrobial) were allowed during the 12-week treatment period.

### 2.5. CADESI-03 Modification

CADESI-03 modification was used to assess lesion severity. The severity of erythema, lichenification, excoriations, and alopecia was assessed at 33 body sites using a scale from 0–5 (0 = none, 1 = mild, 2-3 = moderate, and 4-5 = severe). In CADESI-03, the severity of erythema, lichenification, excoriation, and alopecia was assessed at 62 body sites by using a scale from 0 to 5 [8]. CADESI-03 modification was simplified to assess each symptom at 33 body sites. Total score of CADESI-03 modification was 660 while that of CADESI-03 was 1240. Dogs were scored 4 times during the study: 4 weeks prior to therapy and at week 0, week 6, and week 12 of therapy. The same investigator performed all assessments.

### 2.6. Statistical Analysis

Statistical analysis was performed by use of paired *t*-tests with SAS 8.2 software. Statistical significance was defined as *P* < 0.05. In this study, the mean data for total scores and subscores were pooled at each time point and compared to the other assessment periods.

## 3. Results

**Figure 1:**
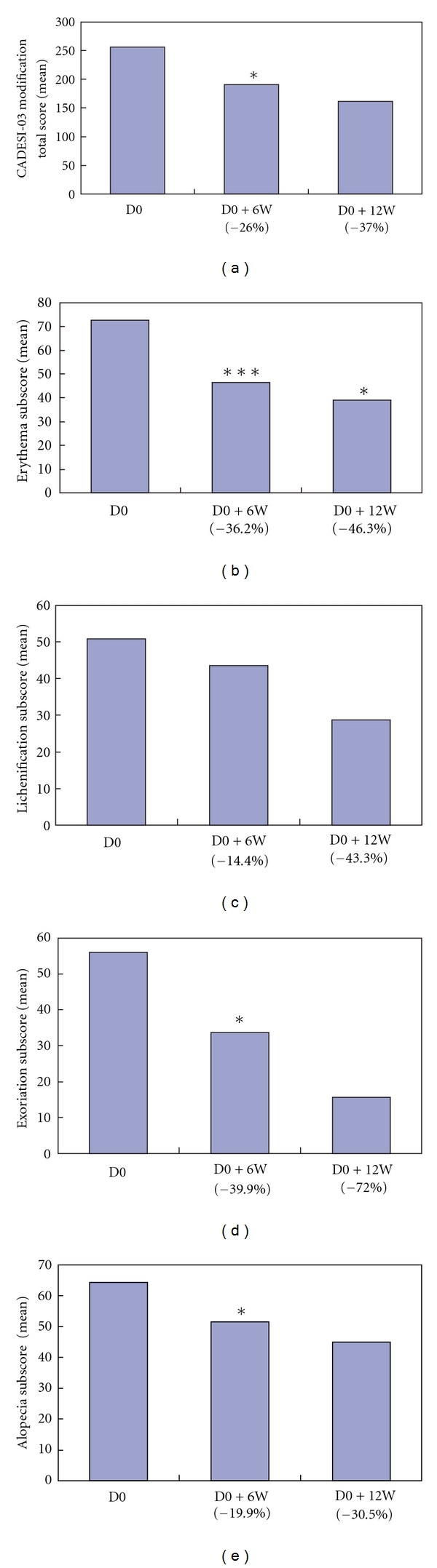
(a) CADESI-03 modification total score improved 6 weeks (D0 + 6W) and 12 weeks (D0 + 12W) after starting treatment (D0) with topical SLC. Results are expressed as mean, *n* = 7. *P < 0.05, compared with D0 score. Number below the abscissa axis indicates average difference rate (%) from D0. (b) Erythema improved dramatically during the trial period. Results are expressed as mean, *n* = 7. *P < 0.05, ***P < 0.005, compared with D0 score. Number below the abscissa axis indicates average difference rate (%) from D0. (c) Mean lichenification subscore. Results are expressed as mean, *n* = 7. Number below the abscissa axis indicates average difference rate (%) from D0. (d) Excoriation improved during the trial period. Results are expressed as mean, *n* = 7. *P < 0.05, compared with D0 score. Number below the abscissa axis indicates average difference rate (%) from D0. (e) Alopecia improved during the trial period. Results are expressed as mean, *n* = 7. *P < 0.05, compared with D0 score. Number below the abscissa axis indicates average difference rate (%) from D0.

**Figure 2:**
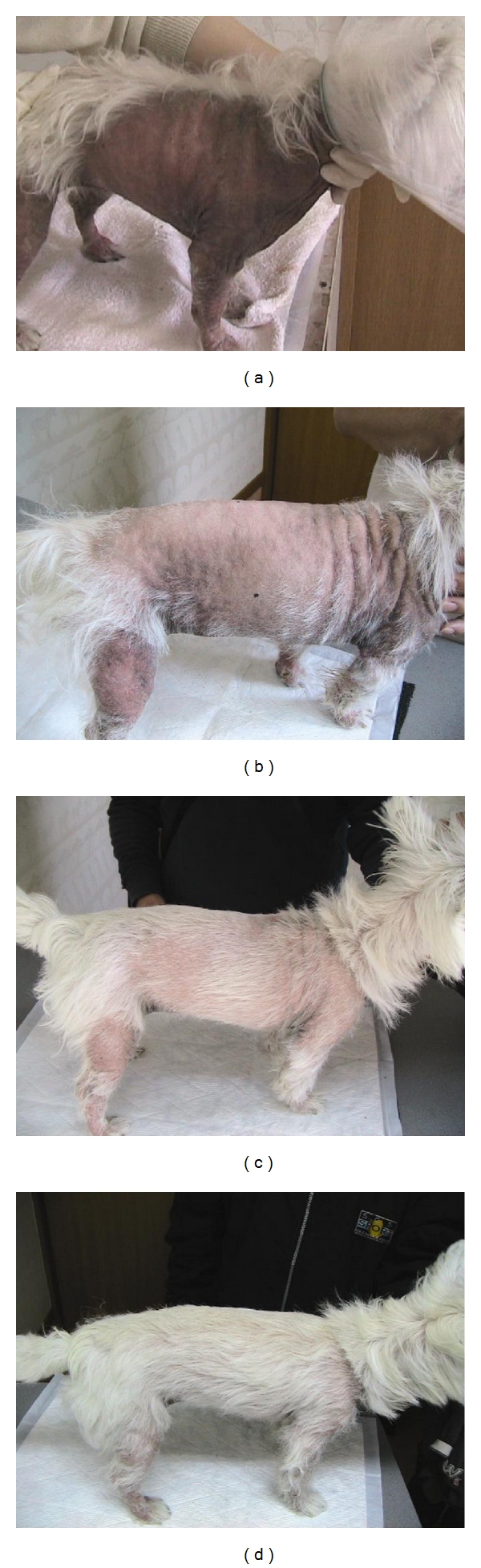
Clinical characteristics of Dog 5 at start of the treatment (D0), 6 weeks after D0, and 12 weeks after treatment are shown in Figures 2(a), 2(b), and 2(c), respectively. Progressive changes in disease severity were seen in the 12-week trial period. The dog was treated continuously after the completion of the trial. Clinical symptoms improved 18 weeks after D0 (Figure 2(d)).

Neither the owners nor the investigators reported any adverse reactions to the application of the SLC therapy. Eight dogs were entered into the study; however, 1 dog was not included in the data analysis, because of a significant increase in pruritus at the start of the study. The owner had discontinued immunotherapy just after starting the study; this was a deviation from the study protocol and the dog was excluded (Dog 6). Clinical data are summarized in Table 1. Female dogs were overrepresented in the study. Dogs ranged in age from 2–12 years (mean 6.75 years). No significant change in each subscore was found between the 4-week pre-treatment assessment and week 0 (*P* > 0.05). When CADESI-03 modification total scores at week 0 were compared to those at 6 weeks after treatment, a significant decrease in scores was noted (26.0%, *P* < 0.05). No significant difference was found between CADESI-03 modification total score at week 6 and week 12. Erythema was the parameter most reduced in the trial period (week 6: 36.2%, *P* < 0.005; week 12: 46.3%, *P* < 0.05) whereas the lichenification score was reduced, but not significantly (week 6: 14.4%, *P* > 0.05; week 12: 43.3%, *P* > 0.05). There was a decrease in excoriation subscore (week 6: 39.9%, *P* < 0.05; week 12: 72.0%, *P* > 0.05) and alopecia subscore (week 6: 19.9%, *P* < 0.05; week 12: 30.5%, *P* > 0.05) from week 0 to week 6 or week 12 (Figures 1(a) to 1(e)). No significant difference of each subscore was found between week 6 and week 12. Progressive changes in Dog 5 over the 12-week period are shown in Figures 2(a) to 2(d).

## 4. Discussion

It is difficult to demonstrate the clinical efficacy of the SLC spot-on treatment in atopic dogs in clinical practice, because topical lipid applications are not used as monotherapy. These formulations are intended to be used as adjunct therapies in combination with other anti-inflammatory drugs, antimicrobial drugs, and immunotherapy as part of the global approach to the management of canine AD. In this study, the investigators found no change in disease severity attributed to the underlying anti-allergic treatment (allergen-specific immunotherapy or oral pentoxifylline administration) in the month prior to inclusion, based upon the unchanged CADESI-03 modification subscores. Immunotherapy was continuously performed for 7 dogs during 36, 31, 34, 7, 14, 6, and 31 months for Dogs 1, 2, 3, 4, 5, 6, and 7, respectively. HDM for Dogs 1, 2, 3, 5, 6, 7, and 8 and cotton linters for Dog 4 were identified as the reacting antigen by skin test for intracutaneous reactivity. Because all dogs were affected with nonseasonal AD, it was considered that the treatment period from September to December had no influence on the skin test result. 

During the 12-week treatment period, however, the total clinical score progressively decreased; decreasing scores are associated with an improvement in clinical signs. The change in the scores for excoriations and erythema was the most dramatic. Because the only change in treatment over the 12 weeks was the addition of the SLC spot-on therapy, these results suggest that topical lipids may play a role in the reduction of inflammation. Piekutowska et al. suggested that SLC spot-on contributed to the formulation of an improved epidermal barrier, as shown by EM [5]. Repair of epidermal barrier function presumably lead to the reduction in inflammation seen in this study. 

In the study reported here, the maximum decrease in the erythema subscore was noted by week 6; in contrast, there was a continued decrease in subscores for excoriations and alopecia. Erythema is a hallmark of inflammation. If inflammation is stabilized, it follows that self-trauma will decrease. The continued decrease in the scores for alopecia is most likely related to the lack of self-trauma and regrowth of hair. The lack of further reductions in the CADESI-03 subscore between week 6 and week 12 of topical SLC treatment is similar to results of a study in people with AD using a topical ceramide-based emollient [3]. Interestingly, in the human study, a subsequent reduction in the frequency of use of the ceramide emollient was associated with a reincrease of the clinical score [3].

Topical lipid applications are not effective as monotherapy, nor are they designed for this use. In people with AD, it has been shown that routine use of a topical physiologic lipid emollient can delay the need for topical glucocorticoid therapy [9]. Our findings are encouraging, but double-blinded placebo-controlled or cross-over studies are needed to confirm our results. In addition, the drug-sparing effects of SLC applications also deserve further investigation.

## 5. Conclusions

In conclusion, the results of this study suggest that topical SLC application is beneficial when used in the management of canine AD.

## Figures and Tables

**Table 1:** Characteristics of dogs at enrollment in the study.

No.	Age at entry in the study (months)	Age at onset of AD (months)	Breed	Sex	Reacting antigen	Combination therapy	
						Immunotherapy (duration (months))	Immunomodulating drugs
1	96	12	Welsh Corgi Pembroke	Mc	HDM	+ (36)	Pentoxifylline
2	96	36	Maltese	Fs	HDM	+ (31)	Pentoxifylline
3	144	36	Shiba inu	Fs	HDM	+ (34)	
4	24	12	Chihuahua	Fs	Cotton Linters	+ (7)	
5	36	12	WHWT	Fs	HDM	+ (14)	
6	36	12	Pug	Fs	HDM	+ (6)	
7	108	24	Shiba inu	F	HDM	+ (31 )	Pentoxifylline
8	108	24	Yorkshire Terrier	F	HDM	—	

WHWT: West Highland White Terrier, Mc: castrated male, Fs: spayed female, F: female, HDM: House Dust Mite Mix (*Dermatophagoides farinae*, *Dermatophagoides pteronyssinus*).

+: immunotherapy administration. —: no immunotherapy.
